# The Design, Synthesis, and Evaluation of the Biological Activity of Hydroxamic Derivatives of Sorafenib

**DOI:** 10.32607/actanaturae.27566

**Published:** 2025

**Authors:** A. A. Kleymenova, I. A. Abramov, Ya. V. Tkachev, P. S. Galeeva, V. A. Kleymenova, N. F. Zakirova, S. N. Kochetkov, M. V. Kozlov

**Affiliations:** Engelhardt Institute of Molecular Biology, Russian Academy of Sciences, Moscow, 119991 Russia; Sechenov First Moscow State Medical University (Sechenov University), Moscow, 119991 Russia

**Keywords:** sorafenib, vorinostat, protein tyrosine kinases, zinc-dependent histone deacetylases, antiproliferative activity, hybrid inhibitors

## Abstract

Sorafenib is a multiple tyrosine kinase inhibitor that is used in the treatment of liver and renal cancers. We synthesized the hydroxamic derivatives of sorafenib bearing the pharmacophore elements of zinc-dependent histone deacetylase inhibitors. We uncovered that suppression of cancer cell proliferation by the synthesized hybrid inhibitors critically depends on the structure of the “deacetylase” element.

## INTRODUCTION

**Fig. 1:**
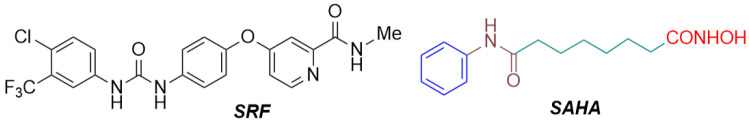
The structures of sorafenib (SRF) and vorinostat (SAHA) with highlighted pharmacophore elements: cap (blue), connecting unit (CU, brown), linker (green), and zinc-binding group (ZBG, red)

Hepatocellular carcinoma (HCC) is one of the most heterogeneous, intractable type of cancer [1]. Sorafenib (***SRF***, *Fig. 1*), a multipotent inhibitor of protein tyrosine kinases (PTKs) – e.g. signaling RAF kinase, VEGFR and PDGFR tyrosine kinases, and others – has proven to be a first-line drug for the treatment of advanced HCC stages [2]. However, longterm use of sorafenib becomes ineffective due to acquired or inherited resistance in some transformed hepatocytes [3].

The combined use of sorafenib with multipotent zinc-dependent histone deacetylase (HDAC) inhibitors is a promising strategy in the treatment of HCC, because many HDAC inhibitors demonstrate not only antiproliferative activity on their own, but also a synergistic effect in combination with sorafenib [4]. For example, the combination of sorafenib with vorinostat (***SAHA***, *Fig. 1*) effectively initiates apoptosis in hepatoma cells [5] and the combination with valproic acid (VPA) significantly delays the development of resistance [6]. In contrast to the combined use of two drugs, monomolecular hybrids boast more predictable pharmacokinetic and pharmacodynamic parameters, including metabolism and bioavailability. In addition, their use ensures the simultaneous activation of several antitumor mechanisms in the tumor site and in the required optimal ratio [7]. Thus, the development of PTK/HDAC hybrid inhibitors seems to be a very promising and justified area of research.

The pharmacophore of histone deacetylase inhibitors (HDACi) comprises four elements: (i) a zinc-binding group (ZBG), (ii) a linker occupying the active site ‘lysine channel’ that leads to the catalytic zinc ion, (iii) a connecting unit (CU), and (iv) an aromatic/heterocyclic fragment (cap) responsible for recognizing the surface of the enzyme’s active site at the entrance to the ‘lysine channel’ [8]. We synthesized new hybrid inhibitors – hydroxamic derivatives of sorafenib – bearing the pharmacophore elements of zinc-dependent histone deacetylase inhibitors. We investigated the antiproliferative activity of the produced compounds and the class selectivity of HDAC inhibition.

## EXPERIMENTAL PART

In this study, we used the following compounds: aminocaproic acid, 4-(aminomethyl)-benzoic acid, diazabicycloundecene (DBU), 1,1’-carbonyldiimidazole (CDI), hydroxylamine hydrochloride, a 50% aqueous hydroxylamine solution, and hydrazine hydrate (Sigma-Aldrich, USA); ethyl ester of 4-aminobenzoic acid (Acros Organics, USA), bis(2-oxo-3-oxazolidinyl)  phosphinic chloride (BOP-Cl) (LEAPChem, China); 4-formyl-N-hydroxybenzamide was synthesized according to [9]. Column chromatography was performed using the Kieselgel silica gel, 0.060–0.200 mm, (Acros Organics); elution systems are provided in the text. TLC was performed on Kieselgel 60 F254 plates (Supelco, USA). NMR spectra (δ, ppm; *J*, Hz) were acquired on an Avance III spectrometer (Bruker, Germany) with an operating frequency of 300 MHz for 1H-NMR (internal standard: Me4Si; solvent: DMSO-*d6*), 100.6 MHz for 13C NMR (with suppression of carbon-proton interaction; solvent: DMSO-*d6*), and 282 MHz for 19F NMR (solvent: DMSO-*d6*). Chemical shifts are provided in parts per million, and spin-spin coupling constants (SSCCs) are expressed in Hz. 1H NMR NOESY and ROESY spectra were measured in dry DMSO-*d6*. The mixing time used for NOESY spectra was specifically selected to maximize the intensity of dipole cross peaks (0.25 s).

**Synthesis of the hydroxamic derivatives of sorafenib* SRF-CHA*, *SRF-BHA*, *SRF-THA*, and *SRF-H-BHA***

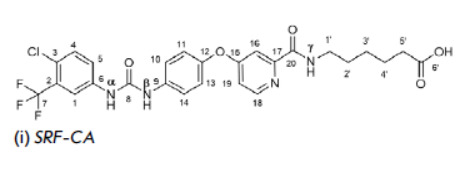



*6-(4-(4-(3-(4-chloro-3-(trifluoromethyl)phenyl)ureido) phenoxy)picolinamide)hexanoic acid (SRF-CA).* A mixture of 466 mg (1 mmol) of a sorafenib carboxylic acid methyl ester (*SRF-ME*) [10], 262 mg (2 mmol) of aminocaproic acid, and 383 mg (2.5 mmol) of DBU in 10 mL of MeOH was stirred under boiling conditions for 6 h. The mixture was cooled to room temperature, diluted with 10 mL of H_2_O, neutralized with HCl (1 : 1) to pH 5–6, and cooled at 10°C for 18 h. The precipitate formed was triturated, filtered, washed with H_2_O, and air-dried. The product was isolated by chromatography on a silica gel using a CHCl_3_/EtOH (10 : 1) mixture as an eluent. The collected fractions were evaporated, and the residue was dissolved in 3 mL of CHCl_3_ and cooled at 10°C for 18 h. The resulting precipitate was filtered, washed with CHCl_3_ and air-dried, finally yielding 400 mg (71%) of *SRF-CA*. 1H NMR (DMSO-*d6*): δ 11.94 (1H, s, OH), 9.20 (1H, s, NHα), 8.97 (1H, s, NHb), 8.76 (1H, t,* J *6.0, NHγ), 8.51 (1H, d, *J *5.6, H18), 8.12 (1H, d, *J *2.1, H16), 7.73–7.53 (4H, m, H4, H10, and H14, H19), 7.40 (1H, d, *J *2.5, H1), 7.24–7.08 (3H, m, H5, H11, and H13), 3.26 (2H, q, *J *6.5, H1′), 2.19 (2H, t, *J *7.3, H5′), 1.65–.43 (4H, m, H2′ and H4′), 1.36–.20 (2H, m, H3′). 13C NMR (DMSO-*d6*): δ 174.87 (C6′), 166.46 (C15), 163.60 (C20), 152.96 (C8 or C17), 152.94 (C8 or C17), 150.77 (C18), 148.35 (C12), 139.80 (C9), 137.51 (C6), 132.44 (C4 or C5), 127.21 (q, *J *30.3, C2), 123.57 (C4 or C5), 123.29 (q,* J *273, C7), 122.85 (C3), 121.89 (C10 and C14), 121.00 (C11 and C13), 117.33 (q, *J *5.5, C1), 114.53 (C19), 109.24 (C16), 39.17 (C1′), 34.04 (C5′), 29.30 (C2′), 26.41 (C3′), 24.68 (C4′). 19F NMR (DMSO-*d_6_*): δ -61.47 (CF_3_).

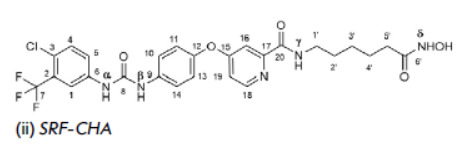



*4-(4-(3-(4-chloro-3-(trifluoromethyl)phenyl)ureido) phenoxy)-N-(6-(hydroxyamino)-6-oxohexyl)picolinamide (SRF-CHA). *A solution of 363 mg (0.643 mmol) of *SRF-CA *in 0.7 mL of DMF was combined with 115 mg (0.71 mmol) of CDI. After 1 h 40 min, 70 mg (1.00 mmol) of hydroxylamine hydrochloride was added, stirred until dissolved for 10 min, and left for 2 h. The reaction mixture was diluted with 3.5 mL of H_2_O and cooled at 10°C for 18 h. The supernatant was decanted, and the precipitated oil was triturated in 7 mL of cold water until the formation of a loose sediment, filtered, and dried in air. The product was isolated by chromatography on a silica gel using a CHCl_3_–EtOH (first 7.5 : 1 and then 5 : 1) mixture as an eluent. The collected fractions were evaporated to yield 262 mg (70%) of *SRF-CHA*. 1H NMR (DMSO-*d6*): δ 10.30 (1H, s, NHd), 9.23 (1H, s, NHα), 9.01 (1H, s, NHb), 8.75 (1H, t, J 5.9, NHγ), 8.63 (1H, s, OH), 8.51 (1H, d, *J *5.5, H18), 8.13 (1H, s, H16), 7.74–7.54 (4H, m, H4, H10 and H14, H19), 7.39 (1H, d, J 2.3, H1), 7.22–7.12 (3H, m, H5, H11, and H13), 3.25 (2H, q, *J *6.6, H1′), 1.94 (2H, t, *J *7.3, H5′), 1.61–.41 (4H, m, H2′ and H4′), 1.34–.17 (2H, m, H3′). 13C NMR (DMSO-*d6*): δ 169.57 (C6′), 166.47 (C15), 163.60 (C20), 152.95 (C8 and C17), 150.77 (C18), 148.35 (C12), 139.79 (C9), 137.51 (C6), 132.44 (C4 or C5), 127.21 (q, *J *30.7, C2), 123.58 (C4 or C5), 123.29 (q, *J *273, C7), 122.85 (C3), 121.89 (C10 and C14), 121.00 (C11 and C13), 117.33 (q, *J *5.5, C1), 114.54 (C19), 109.24 (C16), 39.21 (C1′), 32.67 (C5′), 29.34 (C2′), 26.49 (C3′), 25.33 (C4′). 19F NMR (DMSO-*d6*): δ -61.47 (CF3).

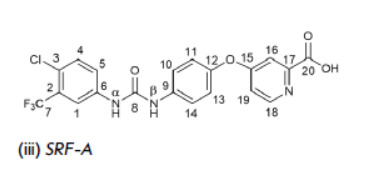



*4-(4-(3-(4-chloro-3-(trifluoromethyl)phenyl)ureido) phenoxy)picolinic acid (SRF-A). *0.67 g (12 mmol) of KOH was dissolved in 12 mL of a THF–MeOH–H_2_O (1 : 1 : 1) mixture, and 2.32 g (5 mmol) of *SRF-ME* was added with stirring in two equal parts over 10 min, and, after dissolution of the starting compound, the mixture was left to rest at room temperature for 1 h. The reaction mixture was diluted with 12 mL of H_2_O and neutralized with HCl (1 : 1) to pH ≈ 1.5. The precipitate was triturated, another 12 mL of H_2_O was added, and the mixture was cooled at 10°C for 1 h. The precipitate was filtered, washed with H_2_O, and air-dried to yield 2.20 g (97%) of *SRF-A*. 1H NMR (DMSO-*d6*): δ 9.29 (1H, s, NHα), 9.06 (1H, s, NHb), 8.58 (1H, d, *J *5.7, H18), 8.13 (1H, d,* J *2.4, H16), 7.70–7.56 (4H, m, H4, H10 and H14, H19), 7.44 (1H, d, *J *2.5 H1), 7.24–7.13 (3H, m, H5, H11 and H13). 13C NMR (DMSO-*d6*): δ 166.47 (C20), 165.85 (C15), 152.96 (C8), 151.19 (C18), 150.86 (C17), 148.20 (C12), 139.81 (C9), 137.65 (C6), 132.44 (C4 or C5), 127.21 (q, *J *30.5, C2), 123.54 (C4 or C5), 123.28 (q, *J *273, C7), 122.83 (C3), 121.84 (C10 and C14), 120.99 (C11 and C13), 117.30 (q, *J *5.5, C1), 115.12 (C19), 112.32 (C16). 19F NMR (DMSO-*d_6_*): δ -61.46 (CF_3_).

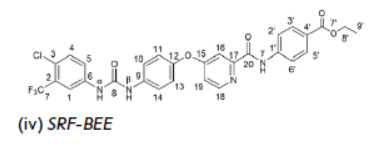



*Ethyl 4-(4-(4-(3-(4-chloro-3-(trifluoromethyl)phenyl) ureido)phenoxy)picolinamide)benzoate (SRF-BEE).* A suspension of 452 mg (1 mmol) of *SRF-A *in 10 mL of a 1 : 1 pyridine–THF mixture was combined with 300 mg (1.18 mmol) of BOP-Cl, stirred for 10 min, and then combined with 230 mg (1.39 mmol) of a *p*-aminobenzoic acid ethyl ester (ABEE). The reaction mixture was stirred at room temperature for 1.5 h, before another 300 mg (1.18 mmol) of BOP-Cl was added. After 10 min, 230 mg (1.39 mmol) of ABEE was added and stirring was continued at room temperature for 1.5 h. Water (30 ml) was added and stirred for 1–1.5 h to form a homogeneous precipitate. The precipitate was filtered and washed with water (thrice, 20 mL each). After air drying, the precipitate was suspended in 10 mL of methanol, filtered, washed with 5 mL of methanol, filtered, and air-dried to yield 442 mg (74%) of *SRF-BEE*. 1H NMR (DMSO-*d6*): δ 10.93 (1H, s, NHγ), δ 9.22 (1H, s, NHα), 9.01 (1H, s, NHb), 8.64 (1H, d, *J *5.6, H18), 8.13 (1H, d, *J *1.9, H16), 8.05 (2H, d, *J *8.8, H3′ and H5′), 7.95 (2H, d, *J *8.7, H2′ and H6′), 7.71–.59 (4H, m, H4, H10 and H14, H19), 7.54 (1H, d, *J *2.5 H1), 7.29–7.16 (3H, m, H5, H11 and H13), 4.30 (2H, q, *J *7.1, H8′), 1.32 (3H, t, *J *7.1, H9′). 13C NMR (DMSO-*d6*): δ 166.70 (C7′), 165.75 (C15), 162.86 (C20), 152.94 (C8), 152.29 (C17), 150.94 (C18), 148.25 (C12), 142.97 (C1´), 139.78 (C9), 137.62 (C6), 132.43 (C4 or C5), 130.46 (C3′ and C5′), 127.20 (q, *J *30.5, C2), 125.48 (C4′), 123.57 (C4 or C5), 123.28 (q, *J *273, C7), 122.85 (C3), 121.88 (C10 and C14), 121.02 (C11 and C13), 120.20 (C2′ and C6′), 117.31 (q,* J *5.4, C1), 115.19 (C19), 110.02 (C16), 60.93 (C8′), 14.63 (C9′). 19F NMR (DMSO-*d_6_*): δ -61.44 (CF_3_).

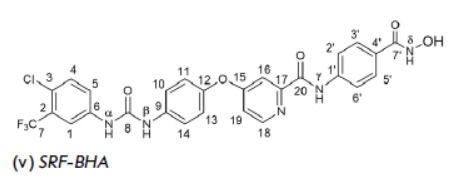



*4-(4-(3-(4-chloro-3-(trifluoromethyl)phenyl)ureido) phenoxy)-N-(4-(hydroxycarbamoyl)phenyl)picolinamide (SRF-BHA). *A solution of 300 mg (0.50 mmol) of *SRF-BEE *in 7.5 mL of a 1 : 2 MeOH–THF mixture was supplemented with 500 mg (7.58 mmol) of NH_2_OH (50%), the mixture was cooled to 0°C, and 56 mg (1.00 mmol) of KOH dissolved in 1 mL of MeOH was added. After 30 min, cooling was ceased and the reaction mixture was left for 18 h. The reaction mixture was cooled to 0°C, and 28 mg (0.5 mmol) of KOH dissolved in 0.5 mL of MeOH was added. After 30 min, cooling was ceased and the mixture was left to rest for 3 h, after which 0.5 mL (8.75 mmol) of AcOH was added, and the mixture was evaporated to half the original volume, before 4 mL of MeOH was added, and the mixture was again evaporated to half its volume. The residue was supplemented with 5 mL of MeOH. The resulting precipitate was triturated, filtered, dried on the filter, successively washed twice with 3 mL of a 2% solution of triethylamine in MeCN, 3 mL of MeCN, and 3 mL of CH_2_Cl2, then air-dried to yield 230 mg (71%) of *SRF-BHA*. 1H NMR (DMSO-*d6*): δ 11.13 (1H, s, NHδ), δ 10.82 (1H, s, NHγ), δ 9.28 (1H, s, NHα), δ 9.07 (1H, s, NHb), δ 8.96 (1H, s, OH), 8.63 (1H, d, *J *5.6, H18), 8.13 (1H, d, *J *2.0, H16), 7.96 (2H, d, *J *8.7, H3′ and H5′), 7.75 (2H, d, *J *8.7, H2′ and H6′), 7.71–.58 (4H, m, H4, H10 and H14, H19), 7.53 (1H, d, *J *2.5, H1), 7.29–.16 (3H, m, H5, H11 and H13). 13C NMR (DMSO-*d6*): δ 166.73 (C7′), 164.35 (C15), 162.86 (C20), 152.97 (C8), 152.43 (C17), 150.94 (C18), 148.26 (C12), 141.18 (C1′), 139.82 (C9), 137.65 (C6), 132.45 (C4 or C5), 128.51 (C4′), 128.03 (C3′ and C5′), 127.21 (q, *J *30.8, C2), 123.59 (C4 or C5), 123.29 (q, *J *273, C7), 122.84 (d, J 1.5, C3), 121.91 (C10 and C14), 121.03 (C11 and C13), 120.16 (C2′ and C6′), 117.33 (q, *J *5.4, C1), 115.16 (C19), 109.93 (C16). 19F NMR (DMSO-*d_6_*): δ -1.44 (CF_3_).

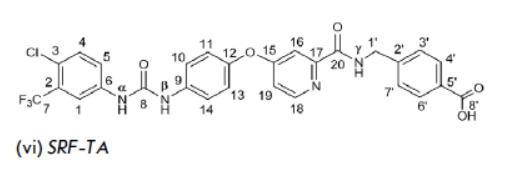



*4-(4-(4-(3-(4-chloro-3-(trifluoromethyl)phenyl)ureido) phenoxy)picolinamide)methylbenzoic acid (SRF-TA).* A mixture of 466 mg (1 mmol) of *SRF-ME*, 302 mg (2 mmol) of 4-(aminomethyl)benzoic acid, and 383 mg (2.5 mmol) of DBU in 6 mL of MeOH was stirred under boiling for 14 h. The reaction mixture was cooled to room temperature, diluted with 15 mL of H_2_O, and neutralized with AcOH to pH ≈ 5–6. The resulting precipitate was triturated and cooled at 10°C for 4 h. The precipitate was filtered, washed with H_2_O, and dried in air. The product was isolated by chromatography on a silica gel using a 5 : 1 CHCl_3_–EtOH mixture supplemented with AcOH (1% of the total volume) as an eluent. The collected fractions were evaporated; the residue was triturated in 10 mL MeOH, filtered, washed with 4 mL of MeOH, and air-dried to yield 269 mg (46%) of *SRF-TA*. 1H NMR (DMSO-*d6*): δ 12.82 (1H, s, OH), 9.44 (1H, t, *J *6.4, NHγ), 9.20 (1H, s, NHα), 8.98 (1H, s, NHb), 8.54 (1H, d, *J *5.6, H18), 8.12 (1H, d,* J *2.4, H16), 7.89 (2H, d, *J *8.3, H4′ and H6′), 7.70–.57 (4H, m, H4, H10 and H14, H19), 7.45–7.37 (3H, m, H1, H3′ and H7′), 7.24–.13 (3H, m, H5, H11 and H13), 4.54 (2H, d, *J *6.3, H1′). 13C NMR (DMSO-*d6*): δ 167.64 (C8′), 166.51 (C15), 164.06 (C20), 152.94 (C8 or C17), 152.67 (C8 or C17), 150.93 (C18), 148.31 (C12), 145.00 (C2′), 139.79 (C9), 137.55 (C6), 132.44 (C4 or C5), 129.83 (C4′ and C6′), 129.78 (C5′),127.75 (C3′ and C7′), 127.21 (q,* J *30.6, C2), 123.58 (C4 or C5), 123.29 (q, *J *273, C7), 122.83 (C3), 121.91 (C10 and C14), 121.01 (C11 and C13), 117.33 (q, *J *5.6, C1), 114.78 (C19), 109.46 (C16), 42.78 (C1′). 19F-NMR (DMSO-*d6*): δ -61.45 (CF3).

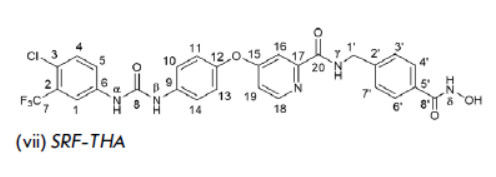



*4-(4-(3-(4-chloro-3-(trifluoromethyl)phenyl)ureido) phenoxy)N-(4-(hydroxycarbamoyl)benzyl)picolinamide (SRF-THA). *A solution of 275 mg (0.47 mmol) of* SRF-TA *in 0.55 mL of DMF was supplemented with 120 mg (0.74 mmol) of CDI. After 1 h 30 min, 120 mg (1.73 mmol) of hydroxylamine hydrochloride was added, the mixture was stirred until dissolution for 10 min and left to rest for 18 h. The reaction mixture was diluted with 7 mL of H_2_O; the precipitate was thoroughly triturated, filtered after 30 min, washed on a filter with 7 mL of H_2_O, and dried in air. The product was isolated by chromatography on a silica gel using a 7 : 1 CHCl_3_–EtOH mixture as an eluent. The collected fractions were evaporated to yield 125 mg (44%) of *SRF-THA*. 1H NMR (DMSO-*d6*): δ 11.14 (1H, s, NHδ), 9.41 (1H, t, *J *6.3, NHγ), 9.23 (1H, s, OH), 9.01 (1H, s, NHα), 8.96 (1H, s, NHb), 8.54 (1H, d, *J *5.6, H18), 8.12 (1H, d, *J *1.8, H16), 7.76–7.55 (6H, m, H4, H10 and H14, H19, H4′ and H6′), 7.41 (1H, d, *J *2.5, H1), 7.36 (2H, d,* J *8.2, H3′ and H7′), 7.26–.12 (3H, m, H5, H11 and H13), 4.50 (2H, d, *J *6.3, H1′). 13C NMR (DMSO-*d6*): δ 166.50 (C15), 164.63 (C8′), 164.01 (C20), 152.94 (C8 or C17), 152.70 (C8 or C17), 150.93 (C18), 148.32 (C12), 143.12 (C2′), 139.79 (C9), 137.54 (C6), 132.45 (C4 or C5), 131.81 (C5′), 127.66 (C2, C4′ and C6′), 127.37 (C2, C3′ and C7′), 127.01 (C2), 123.58 (C4 or C5), 123.29 (q, *J *273, C7), 122.84 (C3), 121.91 (C10 and C14), 121.01 (C11 and C13), 117.33 (q, *J *5.4, C1), 114.77 (C19), 109.44 (C16), 42.72 (C1′). 19F NMR (DMSO-*d_6_*): δ -61.44 (CF_3_).

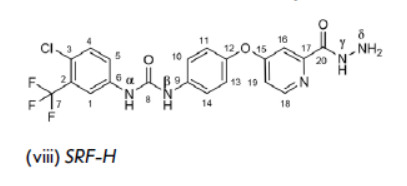



*1-(4-chloro-3-(trifluoromethyl)phenyl)-3-(4-((2-(hydrazinecarbonyl) pyridin-4-yl)oxy)phenyl)urea (SRF-H).* A suspension of 466 mg (1 mmol) of *SRF-ME *in 3 mL of a 2 : 1 MeOH–CH_2_Cl2 mixture was combined with 250 mg (5 mmol) of hydrazine hydrate and stirred for 10 min until the dissolution of the starting compound. After 2 h, 2 mL of MeOH was added and the mixture was evaporated to a thick syrup. After addition of 10 mL of H_2_O, the mixture was triturated until a homogeneous precipitate formed, cooled at 0°C for 1.5 h, filtered, washed with water (twice, 3 mL each), and air-dried to yield 404 mg (87%) of *SRF-H*. 1H-NMR (DMSO-*d6*): δ 9.86 (1H, s, NHγ), 9.19 (1H, s, NHα), 8.97 (1H, s, NHb), 8.48 (1H, d, *J *5.6, H18), 8.12 (1H, d, *J *2.3, H16), 7.72–7.55 (4H, m, H4, H10 and H14, H19), 7.38 (1H, d, *J *2.5 H1), 7.17 (2H, d, *J *8.9, H11 and H13), 7.12 (1H, dd, *J *5.6 and 2.6, H5), 4.56 (2H, s, NHd). 13C NMR (DMSO-*d6*): δ 166.31 (C15), 162.45 (C20), 152.94 (C8), 152.60 (C17), 150.96 (C18), 148.36 (C12), 139.79 (C9), 137.51 (C6), 132.43 (C4 or C5), 127.22 (q, *J *30.8, C2), 123.58 (C4 or C5), 123.29 (q, *J *273, C7), 122.86 (d, *J *1.7, C3), 121.86 (C10 and C14), 121.01 (C11 and C13), 117.34 (q, *J *5.7, C1), 114.33 (C19), 109.25 (C16). 19F NMR (DMSO-*d_6_*): δ -61.45 (CF_3_).

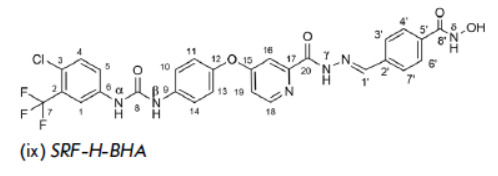



*(E)-4-((2-(4-(4-(3-(4-chloro-3-(trifluoromethyl)phenyl) ureido)phenoxy)picolinoyl)hydrazinoylidene) methyl)-N-hydroxybenzamide (SRF-H-BHA). *A solution of 85 mg (0.515 mmol) of 4-formyl-N-hydroxybenzamide [9] in 3.5 mL of MeOH–CH_2_Cl2, 5 : 2, and 30 μL of AcOHcat was added to a suspension of 233 mg (0.50 mmol) of *SRF-H *in 3 mL of a 2 : 1 MeOH–CH_2_Cl2 mixture, and the mixture was stirred for 5 min until the starting compound had dissolved. After 4 h, the resulting precipitate was filtered, washed successively on a filter with 10 mL of EtOH and 5 mL MeOH, and air-dried to yield 258 mg (84%) of *SRF-H-BHA*. 1H NMR (DMSO-*d6*): δ 12.23 (1H, s, NHγ), 11.27 (1H, s, NHd), 9.25 (1H, s, NHα), 9.08 (1H, s, OH), 9.04 (1H, s, NHb), 8.69 (1H, s, H1′), 8.60 (1H, d, *J *5.6, H18), 8.13 (1H, d, *J *2.2, H16), 7.84 (2H, d, *J *8.4, H3′ and H7′), 7.71 (2H, d, *J *8.4, H4′ and H6′), 7.71–.59 (4H, m, H4, H10 and H14, H19), 7.50 (1H, d, *J *2.5, H1), 7.25–7.18 (3H, m, H5, H11 and H13). 13C NMR (DMSO-*d6*): δ 166.56 (C15), 164.14 (C8′), 160.49 (C20), 152.96 (C8), 152.23 (C17), 151.04 (C18), 148.98 (C1′), 148.27 (C12), 139.80 (C9), 137.62 (C6), 137.23 (C2′), 134.46 (C5′), 132.48 (C4 or C5), 127.93 (C3′ and C7′), 127.47 (C4′ and C6′), 127.21 (q, *J *30.7, C2), 123.63 (C4 or C5), 123.30 (q, *J *273, C7), 122.86 (d, *J *1.3, C3), 121.94 (C10 and C14), 121.05 (C11 and C13), 117.35 (q, *J *5.7, C1), 115.16 (C19), 110.14 (C16). 19F NMR (DMSO-*d_6_*): δ -61.43 (CF_3_).

**Cells, media, and reagents**

In the study, we used the following cell lines: Huh7, Huh7.5, HepG2, and PLC/PRF/5 hepatocellular carcinomas, HCT116 colorectal cancer, SH-SY5Y neuroblastoma, HL60 promyelocytic leukemia, and K562 chronic myeloid leukemia. Differentiated HepaRG cells were produced according to [11]. Sorafenib and vorinostat were purchased from Selleck Chemicals; the fluorogenic substrates Boc-Lys(Acyl)-AMC were prepared as described previously [12].

**Assessment of adherent cell viability**

Adherent cell lines were passaged into 96-well culture plates so that the cell confluence stood at 50–60% 24 h after seeding. The cells were incubated with the investigated inhibitors, at different concentrations, for 48 h, and cell viability was assessed using the Cell Proliferation Kit I (MTT assay) according to the manufacturer’s instructions (Sigma-Aldrich, USA). The optical density of the reduction product, formazan, was measured using a Spark multifunctional plate reader (Tecan Trading, Switzerland) at 544 nm. Each inhibitor concentration was tested at least six times.

**Assessment of differentiated HepaRG cell viability**

Undifferentiated HepaRG cells were passaged into 96-well culture plates (~5 × 10^4^ cells per well) and incubated as described previously [11]. After achieving 100% confluency, the cells were subjected to differentiation. For this purpose, the plates with the cells were kept for 14 days, with the medium changed once every 7 days, then kept in a medium containing 1.8% DMSO (Sigma) for 14 days, with the medium changed once every 7 days. Upon completion of differentiation (28 days), the medium was replaced with a medium containing 1.8% DMSO and the test compounds at the desired concentrations and incubated for 72 h. Cell viability was assessed using the MTT test as described above. Each inhibitor concentration was tested at least eight times.

**Assessment of cell viability in suspension culture**

A cell suspension was passaged into 96-well culture plates (~1.5 × 10^4^ cells per well). After 24 h of seeding, the cells were incubated with the investigated inhibitors in different concentrations for 48 h. Then, 10 μL of a resazurin solution in PBS (2 mg/mL) was added and the cells were kept in a CO_2_ incubator for 4 h. Fluorescence of the reduction product, resafurin, was measured using a Spark multifunctional plate reader (Tecan Trading, Switzerland) at wavelengths of 571_ex_/584_em_ nm. Each inhibitor concentration was tested at least six times.

**Cell-based system for testing the potency and selectivity of HDAC inhibition**

HCT116 cells were passaged into 96-well culture plates so that the cells became 70–80% confluent 24 h after seeding. These cells were incubated with the investigated inhibitors at different concentrations for 24 h. Then, three-quarters of the volume was removed from each well and replaced with the same volume of a cell medium containing both the inhibitor at the same concentration and one of the three substrates; i.e., Sub^Ac/Pro/Tfa^, at a concentration of 30 μM. After an additional 4-h incubation, aliquots of the culture fluid were transferred to a fluorescence assay plate (SPL Life Sciences, Republic of Korea), diluted 2-fold with a trypsin solution (2 mg/mL in Tris- HCl buffer, pH 8.0), and incubated at 37°C for 60 min. Fluorescence was measured using a Spark multi-plate reader (Tecan Trading) at 360_ex_/470_em_ nm. The fluorescence intensity in each well was normalized to the cytotoxicity values obtained for the same well. The fluorescence signal value (in RFU) for each concentration of the test compound was calculated using the following formula:

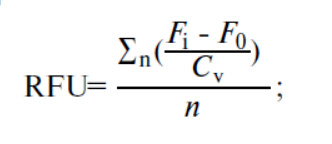



where *F*i is the fluorescence intensity of the sample in the well, *F*0 is the fluorescence intensity in the well with the substrate dissolved in the medium without cells. *C*v is the cell viability, and *n *is the number of replicates.

## RESULTS AND DISCUSSION

**The structural design of hydroxamic derivatives of sorafenib**

**Fig. 2:**
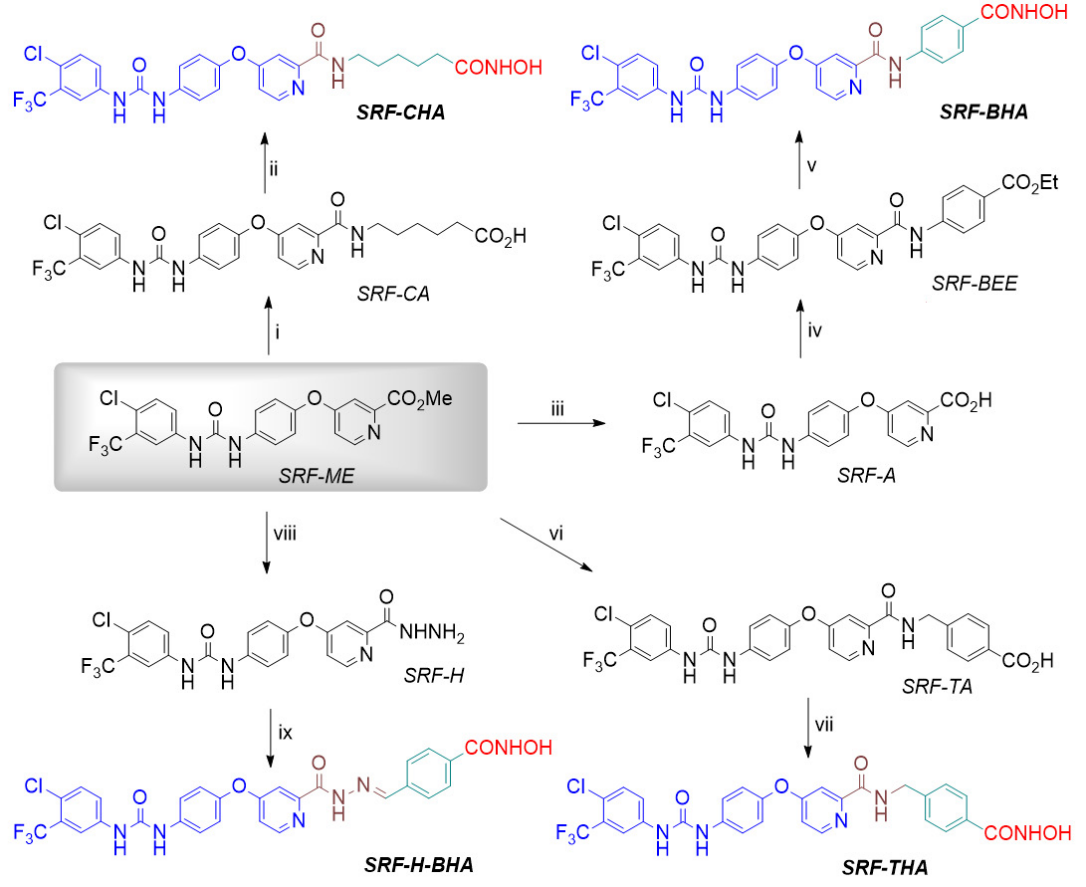
The scheme for the synthesis of the hydroxamic derivatives of sorafenib: SRF-CHA, SRF-BHA, SRF-THA, and SRF-H-BHA: cap (blue), connecting unit (CU, brown), linker (green), and zinc-binding group (ZBG, red). Reagents, conditions, and yield (%): (i) NH_2_(CH_2_)5CO_2_H, DBU, MeOH, D, 6 h, (71%); (ii) CDI, DMF, 2 h, then NH_2_OH·HCl, 18 h, (70%); (iii) KOH, THF/MeOH/H_2_O, 1 h, (97%); (iv) p-NH_2_PhCO_2_Et, BOP-Cl, THF/Py, 3 h, (74%); (v) NH_2_OH, MeOH/THF, 0°C, 0.5 h, then 18 h, (71%); (vi) p-NH_2_CH_2_PhCO_2_H, DBU, MeOH, D, 14 h, (46%); (vii) CDI, DMF, 1.5 h, then NH_2_OH·HCl, 18 h, (44%); (viii) NH_2_NH_2_·H_2_O, MeOH/CH_2_Cl2, 2 h, (87%); (ix) p-(CHO)-PhCONHOH, AcOHcat, MeOH/CH_2_Cl2, 4 h, (84%)

Upon designing hybrid inhibitors (HIs), we were guided by the desire to maximally preserve the structure of sorafenib as a known strong ‘kinase’ component and to use hydroxamic acid moieties, *n*-hexanoic and benzoic acids, characteristic of highly effective HDACi in the ‘deacetylase’ component (*Fig. 2*). In order to meet both requirements, we chose the picolinamide moiety of sorafenib as the docking site for the ‘kinase’ and ‘deacetylase’ components. According to crystallography data [13], this moiety is exposed to the exit from the binding site of sorafenib with the B-RAF kinase active center. We presumed that the ‘deacetylase’ fragment of the hybrid inhibitor, a linker-ZBG, would not create steric hindrances in the interaction with B-RAF.

**Synthesis of the hydroxamic derivatives of sorafenib**

According to the synthesis scheme (*Fig. 2*), the starting compound for the production of all HIs was the sorafenib carboxylic acid methyl ester (*SRF-ME*), whose preparation was described previously [10]. This picoline ester was found to be substantially activated, to the point that the formation of an amide bond with the amino group of ε-aminocaproic and 4-(aminomethyl) benzoic acids was achieved by boiling in methanol in the presence of a strong base (DBU). The resulting carboxylic acids, *SRF-CA *and *SRF-TA*, were converted into the corresponding hydroxamates,*** SRF-CHA ***and ***SRF-THA***, by treatment with CDI and hydroxylamine hydrochloride as described previously [14].

The high reactivity of *SRF-ME *enabled the production of sorafenib carboxylic acid *SRF-A *via mild alkaline hydrolysis in a virtually quantitative yield, similar to [15]. But we had significantly simplified the isolation procedure. Amidation of *SRF-A *with ethyl* p*-aminobenzoate in the presence of the condensation agent BOP-Cl yielded the intermediate ester *SRFBEE* that was used to prepare the target hydroxamate*** SRF-BHA ***by hydroxyaminolysis (*Fig. 2*).

**Fig. 3:**
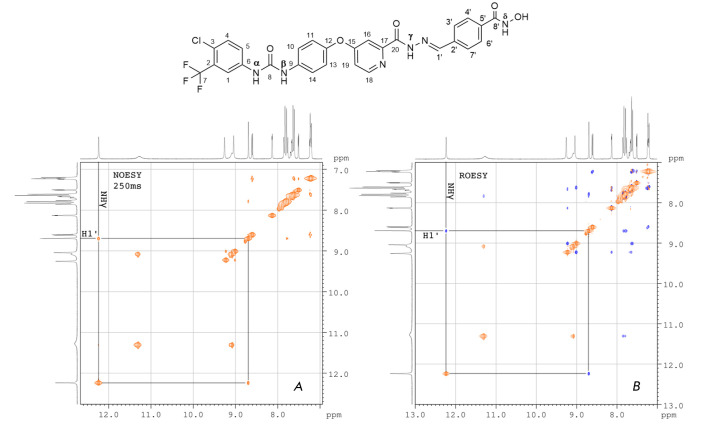
The molecular structure of the (E)-isomer of SRF-H-BHA with atomic numbering as well as two-dimensional 1H spectra (A) NOESY at the mixing time of 0.25 s and (B) ROESY, 8 mg of SRF-H-BHA in DMSO-d6. Cross-peaks between the picolinoylhydrazone proton NHγ and proton H1′ at a double bond are shown. NOESY – Nuclear Overhauser Effect Spectroscopy, ROESY – Rotating frame Overhauser Effect Spectroscopy

Hydrazinolysis of *SRF-ME *proceeded as smoothly as in [10], but somewhat faster. The resulting sorafenib carboxylic acid hydrazide *SRF-H *was used in a click reaction with 4-formyl-N-hydroxybenzamide [9] to yield the required ***SRF-H-BHA ***as the (*E*)-isomer of picolinoylhydrazone (*Fig. 2* and *Fig. 3*). It should be noted that all the synthesized hybrid inhibitors retained the N-monosubstituted picolinate amide moiety of sorafenib (PyCONHR) that, according to crystallographic data, interacts with the main chain carbonyl of Cys531 of B-RAF kinase as part of the complex [13].

**Determination of the picolinoylhydrazone configuration**

To confirm that the produced ***SRF-H-BHA ***is an (*E*)- isomer, we measured two-dimensional NOESY correlation spectra [16] (*Fig. 3A*), which revealed an intense positive cross-peak at (12.23, 8.69), which corresponds to the interaction of H1′ and NHγ protons. Since the molecular weight of the substance occurs in the range between 0.5 and 1.0 kDa, where the nuclear Overhauser effect (NOE) changes its sign (ωτc ~ 1), and the observed dipole-ipole cross peaks cannot generally be distinguished from the exchange peaks by their sign, we measured the ROESY spectrum [17] (NOE in a rotating coordinate system, *Fig. 3B*), where an intense negative cross peak at (12.23, 8.69) was also observed, which clearly confirmed its dipole-ipole nature. The molecular models of ***SRF-H-BHA ***(data not shown) demonstrate that the distance between H1′ and NHγ protons in the (*E*)-isomer is approximately 0.25 nm, which corresponds to the strong NOE observed in the two-dimensional correlation spectra. At the same time, in the (*Z*)-isomer, it is 0.37 nm and occurs near the experimental detection limit of NEO. Therefore, the probability of detecting intense crosspeaks is negligible.

**Evaluation of the cytotoxicity of sorafenib hydroxamic derivatives**

The cytotoxic effect of the produced inhibitors was first tested on a panel of four human hepatoma cell lines: Huh7, Huh7.5, HepG2, and PLC/PRF/5 (*Table 1*). For the same purpose, differentiated HepaRG cells were used, which, as a surrogate for primary human hepatocytes, are widely used to study the cytotoxic effect of xenobiotics [11]. Sorafenib (***SRF***) and vorinostat (***SAHA***), a class I/IIb HDAC inhibitor, were used as reference compounds (*Fig. 1*).

As seen from the data in *Table 1*, the antiproliferative activity of HIs against hepatoma cell lines significantly depended on the structure of the ‘deacetylase’ component linker. Compared with sorafenib, the extended alkyl linker in the ***SRF-CHA ***molecule was linked to a 3- to 4-fold decrease in the antiproliferative activity. Conversely, ***SRF-BHA ***and ***SRFH- BHA ***carrying a phenyl linker were 1.5- to 2-fold more active. The IC_50_ values of the ***SRF-THA ***inhibitor, which comprises a benzyl linker, were close to those of sorafenib, with an upward and downward bias of less than 50%.

Because differentiated HepaRG cells do not proliferate, the decrease in the MTT signal in this case was obviously due to the cytotoxic effect of the inhibitors. The hydroxamic derivatives, ***SRF-BHA***, ***SRFTHA***, and ***SRF-H-BHA***, and the reference compounds,*** SRF ***and ***SAHA***, had approximately equal cytotoxicity in a narrow range of IC_50_ values = 11.3–14.3 μM, and the ***SRF-CHA ***inhibitor was approximately 5-fold less toxic. Interestingly, the profile of IC_50_ values for the entire set of compounds was largely similar to the test results in proliferating PLC/PRF/5 cells, which suggests similar inhibition mechanisms in both cases. The cytotoxic activity of the produced inhibitors was further investigated on the SH-SY5Y neuroblastoma cell line and two suspension leukemia cell lines: HL60 and K562 (*Table 1*). The ***SRF-CHA*** derivative was shown to be much less active than sorafenib against both neuroblastoma and leukemia, whereas the activity of ***SRF-BHA***, ***SRF-THA***, and ***SRF-H-BHA*** was approximately identical to that of sorafenib. Thus, the dependence of HI antiproliferative activity on the structure of the ‘deacetylase’ component in neuroblastoma and leukemia cell lines was identical to that in hepatoma cell lines.

**Testing the potency and selectivity of histone deacetylase inhibition**

**Table 1:** The antiproliferative/cytotoxic effect of hybrid inhibitors on the cell cultures of hepatoma [···], neuroblastoma [···], promyelocytic, and chronic myeloid leukemia [···]; the incubation time was 48 h; in the case of HepaRG cells, the incubation time was 72 h

Cells	Huh7	Huh7.5	HepG2	PLC/PRF/5	HepaRG	SH-SY5Y	HL60	K562
IC_50_, µM								
SRF Sorafenib	3.87±1.14	4.54±0.59	16.6±2.4	18.0±0.9	13.7±2.6	9.19±2.61	6.34±0.21	9.33±0.02
SRF-BHA	3.63±1.42	2.86±1.09	12.9±6.1	7.69±0.81	12.4±4.8	4.00±0.21	5.08±1.50	4.17±0.27
SRF-THA	5.60±0.40	5.27±0.46	8.80±2.21	12.4±3.4	11.3±3.1	7.15±0.27	8.45±3.15	13.1±1.9
SRF-H-BHA	1.80±0.10	2.47±0.82	9.87±1.34	8.33±2.82	12.4±3.9	3.41±0.85	6.97±2.31	9.08±1.23
SRF-CHA	18.1±2.2	14.6±3.5	77.9±4.1	61.3±2.5	69.5±4.8	39.5±9.9	54.5±1.7	51.6±4.6
SAHA Vorinostat	1.73±0.18	1.89±0.22	1.88±0.19	11.4±2.6	14.3±0.6	1.90±0.08	9.43±2.98	8.74±3.06

1–3 µM

3–10 µM

10–30 µM

30–100 µM

The histone deacetylase inhibitor ***SAHA***, which was used as a control, exerted a strong cytotoxic effect on most of the investigated cell lines (*Table 1*). To assess the relationship between the antitumor activity of the hydroxamic derivatives of sorafenib and the suppression of histone deacetylase activity *in cell*, we assessed residual HDAC activity in the presence of HIs using the s3CTS cellular test system as described previously [12]. The s3CTS signal reflected the level of *in-cell *deacylation of three class-selective fluorogenic histone deacetylase substrates of the general structure Boc-Lys(Acyl)-AMC, where Acyl = propionyl (SubPro, HDACs class I), acetyl (SubAc, HDACs class I and IIb), and trifluoroacetyl (SubTfa, HDACs class IIa). Sorafenib and vorinostat were used as negative and positive controls, respectively, for the test system activity.

**Fig. 4:**
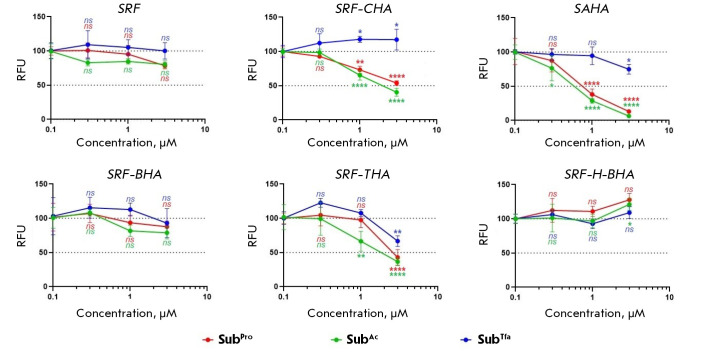
The results of in-cell testing of the selectivity and potency of HDAC inhibition in the presence of sorafenib (SRF), hybrid inhibitors (SRF-CHA, SRF-BHA, SRF-THA, and SRF-H-BHA), and vorinostat (SAHA). Fluorogenic substrates of histone deacetylases: SubPro (HDACs class I), SubAc (HDACs class I and IIb), and SubTfa (HDACs class IIa); RFU is the relative fluorescence units. The statistical significance was calculated using the ANOVA test (GraphPad Prizm 8): ****p < 0.001, ***0.001 < p < 0.01, **0.01 < p < 0.05, *0.05 < p < 0.1, and ns – not significant

***SRF***, ***SRF-BHA***, and ***SRF-H-BHA ***were found not to inhibit *in-cell *histone deacetylase activity up to a concentration of 3 μM (*Fig. 4*). However, ***SRF-CHA*** and ***SAHA***, with the latter a stronger inhibitor, demonstrated the same selectivity as class I and IIb HDAC inhibition. Finally, pan-inhibition of histone deacetylase activity was observed in the presence of ***SRF-THA***. However, the simultaneous decrease in three fluorescent signals observed in this case might point to a malfunction of the s3CTS test system due to additional inhibition of zinc-dependent palmitoyl- CoA thioesterase MBLAC2. It is worth noting that this effect is often observed precisely in the case of selective toluylhydroxamic inhibitors of HDAC6 class IIb, including tubastatin A and nexturastat A [12, 18].

Thus, based on the testing results, only ***SRF-CHA*** and ***SRF-THA ***of the produced four hydroxamic derivatives of sorafenib proved to be histone deacetylase inhibitors (*Fig. 4*). Given the data on antiproliferative activity (*Table 1*), we concluded that the alkyl linker in ***SRF-CHA ***blocked the inhibition of tyrosine protein kinases, and that this effect was only partially compensated by the inhibition of histone deacetylases. A strong negative effect on the activity of sorafenib derivatives with extended alkyl substituents in the picolinamide moiety was noted earlier [10, 19]. The fact that the potency of the antitumor activity of SRF-THA and sorafenib in all the cell lines under study was approximately identical does not contradict previously reported data on the similar values of antiproliferative activity for sorafenib and its N-benzyl derivative [20]. Given these facts, we believe that ***SRF-THA ***may be used to design derivatives that carry a more effective ‘deacetylase’ component.

It is interesting that the antiproliferative activity of both ***SRF-BHA ***and ***SRF-H-BHA ***in most cases significantly exceeded that of sorafenib, although these compounds are not histone deacetylase inhibitors (*Table 1* and *Fig. 4*), which is indirect indication of the enhancement of the ‘kinase’ component in these compounds. As we noted, sorafenib interacts with the main chain carbonyl of Cys531 of B-RAF kinase at the exit from the binding site; in this case, the picolinamide moiety of the inhibitor and the indole ring of Trp530 are parallel to each other and the distance between them is about 4.3 Å [10, 13]. Given this, we suggest the presence of a stacking interaction between the indole ring of Trp530 and the phenyl linker of ***SRF-BHA ***or ***SRF-H-BHA ***because of the mutual coplanarity of both ring systems and their spatial proximity.

## CONCLUSIONS

In this study, by modifying the picolinamide moiety of the inhibitor, we designed and synthesized four hydroxamic derivatives of sorafenib. The structure of all the produced compounds was confirmed by NMR methods. Using *in cell *testing, we showed that only two derivatives, ***SRF-CHA*** and ***SRF-THA***, were able to inhibit HDACs at low micromolar concentrations. Testing the antiproliferative activity of the target compounds in a panel of hepatoma, neuroblastoma, and leukemia cells revealed elevated activity of three compounds:*** SRF-BHA***, ***SRF-THA***, and ***SRF-H-BHA***, comparable or superior to that of sorafenib. ***SRF-THA*** may be used as a parent molecule to develop new hybrid PTK/HDAC inhibitors with high toxicity against tumor cells.

## Abbreviations

PTKs: protein tyrosine kinases
HDACs: zinc-dependent histone deacetylases
B-RAF: signaling tyrosine kinase
SRF: sorafenib
DMSO-d6: deuterated dimethyl sulfoxide
IC50: half maximal inhibitory concentration
AMC: 7-amino-4-methylcoumarin
